# Asymmetric paternal effect on offspring size linked to parent‐of‐origin expression of an insulin‐like growth factor

**DOI:** 10.1002/ece3.3025

**Published:** 2017-05-15

**Authors:** Yolitzi Saldivar Lemus, Jean‐Philippe Vielle‐Calzada, Michael G. Ritchie, Constantino Macías Garcia

**Affiliations:** ^1^ Instituto de EcologíaUniversidad Nacional Autónoma de MéxicoMéxico CityMexico; ^2^ UGA Laboratorio Nacional de Genómica para la Biodiversidad CINVESTAV Irapuato MéxicoIrapuatoMexico; ^3^ School of BiologyUniversity of St. AndrewsSt AndrewsUK

**Keywords:** antagonistic coevolution, Goodeidae, matrotrophy, parental investment, sexual conflict, viviparous fish

## Abstract

Sexual reproduction brings together reproductive partners whose long‐term interests often differ, raising the possibility of conflict over their reproductive investment. Males that enhance maternal investment in their offspring gain fitness benefits, even if this compromises future reproductive investment by iteroparous females. When the conflict occurs at a genomic level, it may be uncovered by crossing divergent populations, as a mismatch in the coevolved patterns of paternal manipulation and maternal resistance may generate asymmetric embryonic growth. We report such an asymmetry in reciprocal crosses between populations of the fish *Girardinichthys multiradiatus*. We also show that a fragment of a gene which can influence embryonic growth (Insulin‐Like Growth Factor 2; *igf2*) exhibits a parent‐of‐origin methylation pattern, where the maternally inherited *igf2* allele has much more 5′ cytosine methylation than the paternally inherited allele. Our findings suggest that male manipulation of maternal investment may have evolved in fish, while the parent‐of‐origin methylation pattern appears to be a potential candidate mechanism modulating this antagonistic coevolution process. However, disruption of other coadaptive processes cannot be ruled out, as these can lead to similar effects as conflict.

## 1 INTRODUCTION

Whenever individuals of different sexes interact, there is the potential for sexual conflict to occur, as the evolutionary interests of both individuals in relation to the outcome of the interaction are normally different (Parker, 1979, 2006). Conflict can arise in relation to current or future mating decisions and also in relation to how much each individual should invest in progeny (Trivers, 1972).

Even if offspring are cared for exclusively by members of one sex (hereafter, as is usually the case, the females), manipulation may still occur if females may be induced to invest preferentially in the brood of the current male, either through sensory manipulation (e.g., Burley, 1986) or by enhancing the ability of the offspring to extract resources from the mother. Viviparity induces an intimate physiological association between embryos and mother, which promotes offspring survival through regular direct provisioning and protection (Blackburn, 1999), while allowing females to adjust the amount and rate of resource delivery (Trexler & DeAngelis, 2003). Viviparous females must trade off current reproductive benefits against survival, future reproduction, and growth (Stearns, 1992). Males do not face the same trade‐offs, as they do not pay the costs but would enjoy greater benefits if the females they mate with increase their investment and produce bigger offspring or larger broods than they would otherwise (Crespi & Semeniuk, 2004; Griggio, Morosinotto, & Pilastro, 2009). Hence, antagonistic coevolution, with males manipulating female investment in offspring, may be expected in species where the amount of maternal investment can be modified after mating.

Sexual conflict can lead to evolutionary divergence (Arnqvist & Rowe, 2002; Chapman et al., 2003) as adaptations that are beneficial for the members of one sex prompt the evolution of countermeasures in the other to mitigate their negative effects (Arnqvist & Rowe, 2002). In the case of viviparous species, excessive male‐induced increments of offspring provision may reduce the mother's lifetime breeding success. Thus, females that develop effective means to resist such manipulation would be favored, leading to a coevolutionary arms race (a form of intergenomic contest evolution, or ICE; Rice & Holland, 1997). Such a process may remain hidden if it leads to resolution of conflict, whereby male adaptations and female counter‐adaptations come into balance (González‐Forero, 2014). Therefore, a powerful method of finding evidence of such antagonism is to make crosses between members of independent populations and species, which are likely to differ in details of the antagonistic coevolution (Rowe, Cameron, & Day, 2003).

This was first observed in deer mice. When females of the monogamous *Peromyscus polionotus* mate with males of polygynous *P. maniculatus*, the size of the hybrids at birth is much larger than that of mice born to intraspecific matings, and they have 5–6 times heavier placentas than those of embryos from the reciprocal cross (Rogers & Dawson, 1970). Subsequent examples have been found in plants (reviewed by Alleman & Doctor, 2000), where several studies provide evidence that in plants with different mating systems, outcrossers can outperform self‐pollinating parents (Brandvain & Haig, 2005), in insects, where the fecundity of honeybees has been shown to be influenced by epigenetic male manipulation (Oldroyd et al., 2014), and in fish, where Schrader and Travis (2008) found that the disruption of maternal‐fetal coadaptation in crosses between populations of *Heterandria formosa* (a highly matrotrophic poeciliid species) results in differential embryo mortality linked to differences in maternal investment (Schrader, Fuller, & Travis, 2013; Schrader, Travis, & Fuller, 2011), and, again using interpopulation crosses, they demonstrated that embryos can influence maternal investment and that investment is traded versus fecundity (Schrader & Travis, 2009).

The best documented example of a mechanism of male epigenetic manipulation of female investment was the finding that the expression of the gene responsible for the synthesis of the insulin‐like growth factor 2 (IGF2) and of its receptor (IGF2R) is epigenetically influenced in mouse embryos (DeChiara, Robertson, & Efstratiadis, 1991). IGF2 is a protein that promotes growth and cellular differentiation during development (Cohick & Clemmons, 1993), and in mammals, it also regulates the placental supply of nutrients and the demand of nutrients by the fetus (Constância et al., 2002). Excess IGF2 in the cell is captured and transported to the lysosomes for subsequent degradation by the cation‐independent mannose‐6‐phosphate receptor, a membrane protein encoded by the gene *igf2r* (Kornfeld & Mellman, 1989), which plays an essential role in regulating normal fetal growth, circulating level of IGF2, and heart development (DeChiara et al., 1991; Lau et al., 1994). In therian mammals, these genes are expressed in a parent‐of‐origin manner. The paternal allele of *igf2* is translated while the maternal allele remains inactivated (DeChiara, Efstratiadis, & Robertson, 1990), and the opposite expression pattern is found in *igf2r*, which is maternally active and paternally silent in artiodactyls, rodents, and marsupials (although it is biallelically expressed in Scandentia, Dermoptera and Primates; Barlow et al., 1991; Killian et al., 2001a). Imprinting of these genes occurs in Therian mammals, but not in monotremes or birds (O'Neill et al., 2000; Killian et al., b).

The *igf2* gene has been found in several fish species—including the Goodeidae (Poeciliidae, Lawton et al., 2005; Cyprinidae, Yuan et al., 2011), where it has been demonstrated to be under positive selection (O'Neill et al., 2007) and is expressed in their embryos. Additionally, matrotrophy, an advanced form of viviparity involving maternal provisioning of embryos through gestation, is present in at least 11 fish families, where it may have evolved independently (Wourms, Grove, & Lombardi, 1988). Theoretically, such viviparity has been considered to be potentially one of the main drivers of population divergence because of the close and particular physiological interactions between mother and embryo that may result in a conflict between them or between both parents over the level of maternal investment (Trivers, 1974; Zeh & Zeh, 2000).

One group of viviparous fish with advanced matrotrophy are the Mexican Goodeidae (Goodeinae, Lombardi & Wourms, 1985a,b). This is a clade of ca. 40 species distributed in 17 or 18 genera (Webb et al., 2004), a ratio of genera to species that suggests rapid speciation. This might be driven by the evolution of viviparity or possibly by the high prevalence of sexual selection, which is itself linked to the extreme sexual asymmetry in parental care that viviparity entails (Macías Garcia, 2014). Asymmetry in parental investment is particularly large in the Goodeinae, in which females nourish their embryos through unique specialized embryonic tissues known as trophotaeniae (Schindler, 2005) for 7–8 weeks (Macías‐Garcia & Saborío, 2004), during which they grow up to 38,700% (Lombardi & Wourms, 1985a). Extended maternal provisioning and a specialized placenta‐like structure make Goodeinae fish potentially good models for the study of antagonistic coevolution of parental allocation of resources to developing embryos, a possibility that has not previously been addressed. We looked for paternal effects on offspring development and size in crosses between populations, a pattern that could be consistent with antagonistic manipulation of offspring development.

## 2 METHODS

### 2.1 Study species

**Figure 1:**
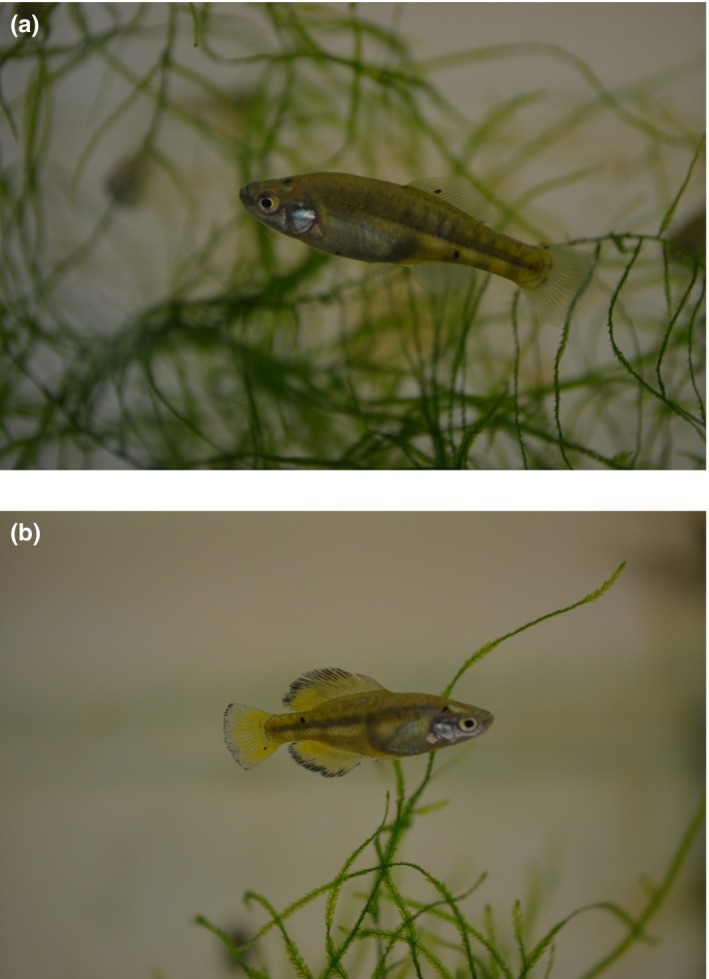
Photograph of a (a) female and (b) male of *G. multiradiatus* from San Matías el Grande population

The Amarillo (Figure 1) is found in water bodies of the upper Lerma River basin, and in limited upland regions of the adjacent Balsas and Pánuco catchments (Gesundheit & Macias‐Garcia, 2005). Males have much larger and colorful median fins than the females, who base their mate choice on these ornaments and on courtship performance (González Zuarth & Macías Garcia, 2006). There has been rapid population divergence (Macías Garcia et al., 2012; Ritchie et al., 2007) and female mate choice often—but not always—leads to premating isolation between populations (González Zuarth & Macías Garcia, 2006; Macías Garcia et al., 2012). For this study, we selected the two populations that are most distant geographically and genetically; Zempoala (Z), a mountain population in the watershed between the southernmost reaches of the Lerma and the Balsas catchments, and San Matías (M), in the Balsas basin, at the northwestern corner of the Amarillo distribution (Macías Garcia et al., 2012). Genetic distance between these populations, based on microsatellite variation, is large (Macías Garcia et al., 2012). If offspring development is subject to some kind of parental antagonistic manipulation, we predicted that offspring size and weight would show paternal effects in crosses between populations. We also explored whether *igf2* shows evidence of a parental effect via parent‐of‐origin methylation patterns.

All methods were carried out in accordance with the guidelines for the treatment of animals in behavioral research and teaching published by Animal Behaviour (https://doi.org/10.1016/j.anbehav.2011.10.031). Fish were kept at the Instituto de Ecología, UNAM.

### 2.2 Interpopulation crosses

Fish were collected under SAGARPA permit DGOPA/01262/040310.0716 and were promptly transported to aquaria at the Instituto de Ecología, UNAM in local water, stress coat, and antiseptics. They were maintained at a 12‐hr day–night cycle, 21°C, and fed SeraVipan™ commercial fish flakes twice a day. New born fish were kept in 80‐L population‐specific aquaria until sex could be determined.

Between 60 and 125 days of age (100 ± 16 days; see De Gasperin & Macías Garcia, 2014), each fish was assigned to one of the following crosses (female–male): (1) M‐M, (2) Z‐Z, (3) M‐Z, and (4) Z‐M. Females were kept with the appropriate males either in one communal 80‐L tank per cross (*n* = 49 females, or 62% of the final sample) or in smaller groups of one or two pairs, but at a comparable density within 20‐L tanks (*n* = 30 females, 38% of the final sample). The distribution of females kept in either condition was similar for all crosses (*x* ^2^ = 1.2, *df* = 3, *p* = .75). Rearing condition, which was entered as a fixed factor in the analysis, had no effect on either brood or offspring size. Stress coat‐treated gravid females were initially weighed once a week using an electronic scale (Ohaus Scout, SC2020) and then every two days as birth became imminent (usually in weeks 7 and 8). We did not measure male length because only about one‐third of the broods (those born to pairs living in isolation) could be assigned to a particular sire.

Female body length (standard length) and width were measured from digital photographs taken the following day, once all offspring had been delivered, using UTHSCSA Image Tool freeware. Individual offspring were measured in the same way as their mothers, but their mass was obtained by weighing the entire brood and then dividing the value by the number of fish. Some females died during or shortly after giving birth but before all her measures were taken; therefore, we ended up with different sample sizes. We entered female length (SL) as a covariate in the analyses (female SL was highly and significantly correlated with female width; *r *=* *.93, *F*_(1,70)_ = 418.7, *p *<* *.0001). We compared breeding performance and brood attributes using mixed models in which each brood was used only once on each analysis (brood size, mean offspring mass, and the ratio of brood mass/female mass before parturition (reproductive allocation; RA; Abrahamson & Gadgil, 1973). Individual offspring SL and width were nested within brood. All our mixed models included female identity as a random factor, female SL and rearing environment as covariates, and female population of origin as one fixed factor; they also included male population of origin and the interaction between male and female population, as these two effects would be indicative of offspring genotype influencing female parental investment (Reznick, 1981; Schrader & Travis, 2009). Reported post hoc probabilities are corrected (Bonferroni) for multiple comparisons. All analyses were performed using NCSS 2007 v. 7.1.21.

### 2.3 Parent‐of‐origin *igf2* expression

A ≈5‐kb fragment of *igf2* was cloned and sequenced using primers adapted from the published sequence of *Ilyodon ameca* (GenBank Accession number DQ337453.1) (see Appendix S1 for details on *igf2* sequencing and SNPs analysis), and screened for SNPs. We used a SNP located in the coding region of fish from Zempoala to evaluate parent‐of‐origin expression of *igf2*. First, we generated several breeding groups, always made of one Z–Z pair, and in some cases an additional female from either Huapango (in the vicinity of San Matías) or Tonatiahua (in the Zempoala lakes National Park), as females from San Matías were temporarily unavailable. Fish within an interpopulation pair were raised together to overcome preferences for intrapopulation partners (De Gasperin & Macías Garcia, 2014). Fish were kept under standardized conditions (see Appendix S1), and each resulting pregnant female and her entire brood were sacrificed around the 7th week of pregnancy, when we collected a fin clipping from the sire and stored the tissues either in absolute ethanol or in RNAlater.

#### 2.3.1 Genotyping of families

**Figure 2:**
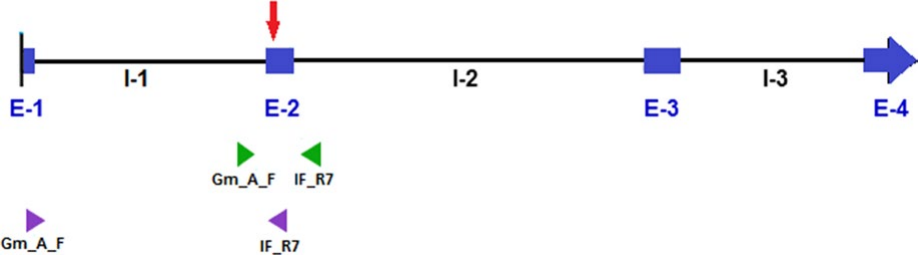
Scheme of *igf2* of *G. multiradiatus*. Blue boxes represent exons, continuous lines introns, and the red arrow shows the approximate location of the selected SNP. Green arrowheads represent the binding site of the primers used for genotyping, and purple arrowheads show the binding site of the primers used for RT‐PCRs.

The *igf2* gene of teleosts is typically composed of four exons and three introns (Juhua et al., 2010). We screened for SNPs from exon 2 (Figure 2) of 22 breeding pairs (36 individuals, as five males were shared by two females and one male by three females). Primers (see Appendix S1) amplified a product of 443 nucleotides that contained exon 2 in its entirety, plus some segments of the adjacent introns.

Genomic DNA was extracted with a homemade protocol (see Appendix S1 for details). The PCR reaction system contained 10 μl of GoTaq Green Master Mix (Promega), 6 μl Milli Q water, 1 μl DMSO, 1 μl I2_F_P (20 pmol/μl), 1 μl I2_R_P (20 pmol/μl), 1 μl DNA, and was exposed to 30 PCR cycles of 95°C for 5 min, 94°C for 30 s, 59°C for 30 s, 72°C for 30 s followed by 10 min at 72°C. PCR products were cloned using TOPO TA Cloning kit (Invitrogen) for electrocompetent cells (TOP10 Electrocomp). Plasmid DNA was extracted following alkaline lysis protocol by Sambrook, Fritsch, and Maniatis (1989). A minimum of 10 clones per individual were sent for sequencing, and sequences were analyzed with BioEdit Sequence Alignment Editor. We genotyped the offspring of families in which we could track the parental alleles (i.e., parents were not homozygous for the same SNP) and screened the brood for heterozygous embryos, as before.

#### 2.3.2 Assessing gene expression through RT‐PCR

Total mRNA of heterozygous offspring was isolated using TRIzol (Invitrogen) and was then reverse transcribed using SuperScript II Reverse Transcriptase and oligodT primers, according to the manufacturer's protocol (Invitrogen). The cDNA was employed as a template for PCR amplification using specific primers. To distinguish the size of the amplified cDNA product from genomic fragments that could be amplified after inefficient DNAse digestion, forward and reverse primers were anchored in exons 1 and 2, respectively. We cloned the fragment as before, analyzed a minimum of 50 clones per individual, and determined which allelic variant (parental allele) had been recovered.

### 2.4 Bisulfite sequencing

Parent‐of‐origin expression effects often occur by genomic DNA methylation, involving the addition of a methyl group to cytosine residues of the dinucleotide CpG (Hendrich & Tweedie, 2003).These can be revealed by treating genomic DNA with bisulfite, which converts cytosine residues to uracil (translated into thymine during sequencing). To determine whether the asymmetric effects on offspring size could be influenced by parent‐of‐origin effects in the methylation state of *igf2*, we took advantage of a heterozygous C/T embryo (P21‐3) that inherited a T allele from its mother and a C allele from its father, and of a heterozygous C/T adult female (P11‐F)—although here we did not know the parental origin of each allele—and analyzed the pattern of 5′ cytosine methylation in a 443‐bp fragment that spanned the SNP site by treating genomic DNA with bisulfite before PCR amplification and cloning.

Bisulfite sequencing was performed as reported in Lim et al. (2015) with minor modifications using DNA from the two individuals mentionned above. Samples of 500 ng of genomic DNA (obtained as above) were bisulfite converted using EZ DNA methylation‐direct kit (Zymo Research), eluted in 30 μl elution buffer, and 1 μl of each aliquot was PCR amplified using primers forward: Forward1 and Forward2 and reverse: Reverse1 and Reverse2 (designed as above; see Appendix S1: Table S2) (95°C for 5 min, 20 cycles of 94°C for 30 s, 59°C for 30 s, 72°C for 30 s each, 72°C for 10 min.). PCR products were gel purified, cloned into pDRIVE cloning vector using Qiagen PCR cloning kit (Qiagen, Valencia, CA), and transformed into DH10B cells before sequencing.

## 3 RESULTS

### 3.1 Interpopulation crosses

**Figure 3:**
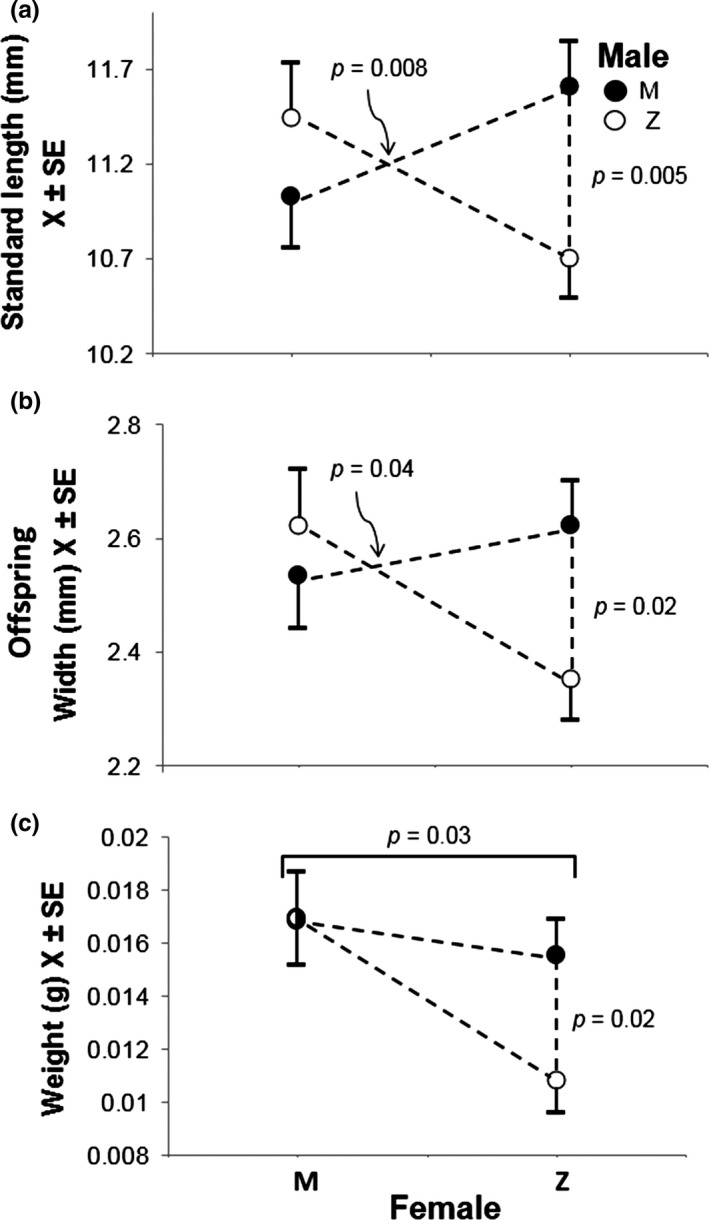
(a) Length (SL), (b) width, and (c) weight of the offspring from intra‐ and interpopulation crosses of adult *G. multiradiatus*. Significant interactions between paternal and maternal (*X*‐axis) origin seen in (a) and (b) are predicted when there is sexual conflict over parental provisioning of embryos. Graphs based on adjusted means to discount the effect of correlated female size

F1 females from Z were smaller than from M (*t *=* *4.57, *df *= 70, *p *<* *.0001; Table 1) but gave birth to larger broods than M females (Bonferroni *F*_(1,66.0)_ **= 6.30, *p *=* *.01; Table 1). Furthermore, Z females did not produce smaller offspring than M females when mated with males of their own population (Z‐Z vs. M‐M; Bonferroni *F*_(1,67.1)_ **= 0.93, *p *=* *1.0; Figure 3a), and when mated with M males, they gave birth to larger offspring than to those produced by Z females mated with Z males (Bonferroni *F*_(1,63.0)_ **= 9.83, *p *=* *.02). Also, we detected a significant interaction between male and female population of origin on offspring size (*F*_(1,64.4)_ = 7.45, *p *=* *.008) as well as a significant effect of the cross (*F*_(3,64.6)_ = 3.7, *p *=* *.02). We observed a similar pattern with offspring width, with no effect of female population (*F*_(1,65.2)_ = 0.94, *p* = .34), and a significant male X female interaction (*F*_(1,65.2)_ **= 4.51, *p* = .04); offspring of Z‐M were wider than those from Z‐Z crosses (Bonferroni *F*_(1,63.5)_ = 7.14, *p *=* *.02; Figure 3b). Neither length nor width of offspring from M females differs between crosses (length, Bonferroni *F*_(1,65.6)_ = 1.17, *p *=* *.57; width, Bonferroni's *F*_(1,66.4)_ = 0.32, *p *=* *1; see Appendix S1: Tables S3 and S4).

**Table 1:** Size and fecundity of F1 females

Variable	Cross											
	M–M			M–Z			Z–M			Z–Z		
	*X*	*SD*	*N*	*X*	*SD*	*N*	*X*	*SD*	*N*	*X*	*SD*	*N*
Mother												
SL (mm)	34.05	5.51	15	34.79	3.68	11	30.32	3.83	17	29.05	4.18	29
W (mm)	8.82	1.6	15	9.35	1.27	11	7.68	1.35	17	7.27	1.11	29
Mass (g)	0.56	0.21	16	0.54	0.16	11	0.36	1.36	17	0.32	0.17	29
Brood size	6.1	4.46	19	5.54	3.38	13	5.72	3.91	18	6.59	3.12	30
RA	0.14	0.08	16	0.15	0.07	11	0.17	0.08	17	0.14	0.06	29
Mean offspring												
SL (mm)	11.2	1.28	16	11.73	0.78	11	11.36	1.13	17	10.3	1.11	28
W (mm)	2.55	0.5	16	2.69	0.29	11	2.56	0.34	17	2.25	0.36	28
Mass (g)	0.17	0.006	16	0.18	0.005	11	0.15	0.008	17	0.1	0.005	29

Reproductive allocation (RA) is the ratio of total brood mass/brood + mother mass (SL, standard length; W, width).

Neonates produced by M females were heavier than those produced by the smaller Z females (*F*_(1,66.0)_ = 5.15, *p *=* *.03; Table 1; Figure 3c). Weight significantly covaried with maternal length (*F*_(1,66.0)_ = 6.21, *p *=* *.015). As above, Z females mated with M males produced heavier offspring than their controls (*F*_(1,66.0)_ = 7.17, *p *=* *.02), although the male X female interaction was not significant.

Zempoala females appear to allocate more resources to offspring production than females from San Matías (although the difference fell short of significance; *F*_(1,52.0)_ = 3.19, *p *=* *.08; see Appendix S1: Fig. S2a) but this apparent difference was not related to the population of origin of the male sire (male × female interaction, *F*_(1,52)_ = 1.12, *p *=* *.29); thus, the interaction between paternal and maternal population of origin on offspring size was not due to a male influence on the female RA.

### 3.2 Parent‐of‐origin *igf2* expression

#### 3.2.1 Genotyping of families

We only found one SNP (C/T) sufficiently frequent to be used as a marker of parent‐of‐origin expression of *igf2*, yet in spite of extensive crosses (*n* = 22 pairs), only three heterozygous offspring were obtained. When cloning the gene from two of these, only the paternal allele was recovered. Although suggestive, our assessment of parent‐of‐origin expression of *igf2* is, consequently, inconclusive given the scarcity of heterozygous fish (see Appendix S1 for details).

### 3.3 Bisulfite sequencing

**Figure 4:**
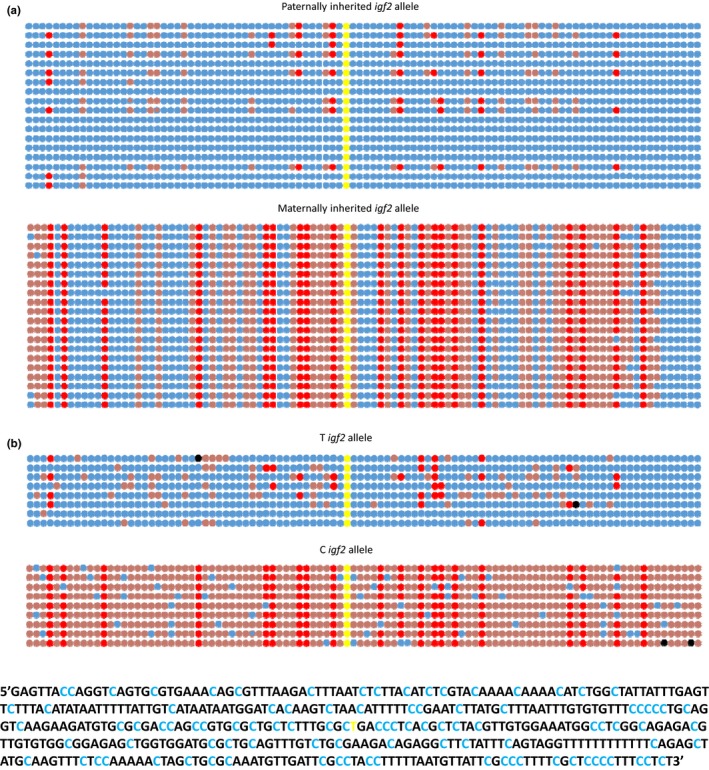
Parent‐of‐origin effects in genomic DNA methylation at the *igf2* gene. 5′–3′ linear representation of cytosines present in a 443‐bp genomic fragment spanning an informative SNP (highlighted in yellow) that allow distinction between maternally and paternally inherited IGF2 gene copies in a) a heterozygous offspring (P21‐3) and b) a heterozygous female (P11‐F); 5′ methylated cytosines in a CpG context are represented by dark red dots, 5′ methylated cytosines in a different context are shown as light red dots, unmethylated cytosines are indicated as blue dots, and cytosines of undetermined methylation status are indicated as black dots. The lineal sequence of the fragment is shown below the graphic depiction of methylation. The cytosines are highlighted in light blue, and the nucleotide of the SNP (C/T) is highlighted in yellow

Thirty‐eight independent fragments of *igf2* from P21‐3 were sequenced: 20 corresponded to the maternally and 18 to the paternally inherited *igf2* copy (*Χ* ^2^ = 0.105, *df* = 1, *p* > .05). Strikingly, 5′‐methylcytosines in a CpG context were only prevalent (i.e., present in >50% of the clones) in sequences representing a maternally inherited *igf2* copy and were virtually absent from copies that were paternally inherited (Figure 4a). Additional cytosines present in non‐CpG positions were also frequently methylated in the maternally inherited *igf2* copy, contributing to a highly contrasting methylation pattern that correlates with the monoallelic expression of *igf2* during embryogenesis.

Seventeen independent sequences from adult female P11‐F were obtained; the eight belonging to one allele were hypomethylated, and the nine sequences of the other allele were hypermethylated (Figure 4b). As with P21‐3, these segregations are not different from 1:1 (*Χ* ^*2*^ = 0.06, *df* = 1, *p* > .05), indicating that the cytosine residue present at position 225 of the amplified fragment (corresponding to the P21‐3 paternally inherited *igf2* copy) is not affected by the bisulfite treatment, allowing for a comparison of the methylation pattern among both alleles.

## 4 DISCUSSION

Here, we demonstrate an interaction between paternal and maternal origin in the size attained at birth by *G. multiradiatus* offspring. This is not the consequence of population differences in female size, nor, apparently, of major differences in reproductive allocation (but see Appendix S1). We also found differences in how resources transferred to their embryos are used in both populations. While offspring size was similar, the smaller females from Zempoala produced more, but lighter, newborn fish than their San Matías counterparts. These patterns are influenced by sire, because Zempoala females, when mated with a male from San Matías, resulted in larger, wider, and heavier offspring than mating with a Zempoala male (Figure 3), further suggesting that the male origin influences offspring growth in this matrotrophic fish through an interaction between the maternal and paternal contributions. We did not find evidence that brood number was influenced by the males, which may signify that females have a somewhat fixed amount of resources to invest or a set number of ova to fertilize in each brood (see Appendix S1: Fig. S2b).

The size of offspring from matings between populations can depart from additive expectations for several reasons. In positive heterosis (hybrid vigor, or simply “heterosis”; Shull, 1908), offspring from both reciprocal crosses would be expected to be similarly larger (or healthier, or fitter) than offspring form the parental populations (e.g., Shikano, Nakadate, & Fujio, 2000), whereas outbreeding depression would cause smaller or less fit offspring in both interpopulation crosses. These effects are expected to be symmetrical, due to either the amelioration of mutational load (Keller & Waller, 2002) or the breakdown of co‐adapted genomes (Templeton et al., 1986) and are thus unlikely explanations for the phenotypic effects seen here (but we note that M‐Z hybrids were somewhat, but nonsignificantly, larger than M‐M offspring). Furthermore, disruption of coadapted complexes in F1 hybrids is usually only seen in one sex (Haldane, 1922), and although we do not have data on the sex of the newborn, we found no male X female effect in the coefficient of variation of offspring size (*F*_(1,66)_ = 0.21, *p* = .65), as would have been expected if Haldane's rule was occurring.

Differences between parental and hybrid phenotypes can also be the consequence of maternal effects if, for instance, females perceive the males from the alternative populations to be more attractive than those from their own, and preferentially invest in offspring of attractive males (Burley, 1988; Gil et al., 1999). This is unlikely to explain our results as females from both localities are reluctant to mate with males from the other population (González Zuarth & Macías Garcia, 2006) unless they are raised together from an early age (De Gasperin & Macías Garcia, 2014). Maternal effects are also an unlikely explanation because only the offspring of Z‐M crosses, and not those from M‐Z, were larger than their controls (Figure 3a).

Breakdown of genetic coadaptations can result in phenotypic effects in interpopulation crosses such as those described here. Genes will have coevolved to function properly in the context of other genes involved in the same processes, giving raise to coadapted clusters of genes that may differ between populations (Wolf, 2013). Crosses between populations may break down such coadapted clusters and generate a diversity of unpredictable phenotypic patterns. Similarly, the details of the necessary coadaptation between mother and embryos may vary between populations and may also be disrupted by interpopulation crosses (Wolf & Brodie, 2009). Disruption of gene coadaptation through outcrossing may also lead to genes been silenced (Ortíz‐Barrientos, Counterman, & Noor, 2007), which might lead to monoallelic expression or to the disruption of genomic imprinting (Wolf, Oakey, & Feil, 2014). Our results are also consistent with expectations derived from sexual conflict (Parker, 1979, 2006). Goodeid matrotrophic viviparity involves a massive, protracted transfer of nutrients to the embryos (Lombardi & Wourms, 1985a,b) that can be co‐opted by males. There is no evidence suggesting that males can influence female investment through sensory stimulation during courtship, but we show evidence that *igf2*, a gene whose overexpression may influence embryonic growth, has a parent‐specific methylation pattern, which suggest a possibly epigenetic parental effect. At present, we cannot distinguish between the co‐adaptation and the conflict hypotheses (see Schrader et al., 2013).

The *igf2* gene encodes insulin‐like growth factor 2 (IGF2) which plays an important role in embryonic development. It is involved in nutrient exchange between mother and embryo (Constância et al., 2002; Reik et al., 2003) and can, therefore, affect the amount of nutrients transferred to the developing offspring. An analysis of nonsynonymous mutations in the mRNA of *igf2* has shown this gene to be under positive selection in several placental cyprinodontiformes (O'Neill et al., 2007), implying that it is involved in the development of matrotrophic fish embryos. O'Neill et al. (O'Neill et al., 2007) inferred that evidence of sustained directional selection on the coding sequence of this gene in matrotrophic cyprinodontiformes amounts to evidence of parent–offspring conflict driving *igf2* evolution. This is plausible, but the argument cannot be compelling unless it is also shown that either 1) an antagonistic gene (e.g., the *igf2r*) has experienced a comparable evolutionary divergence, or 2) that the expression of *igf2* in the embryos follows a parent‐of‐origin pattern (i.e., that there is a bias in embryos to express the paternal allele). We only demonstrated a parent‐of‐origin methylation pattern in the developing embryos, but these data, together with (1) the evidence of *igf2* being expressed in fish embryos (Lawton et al., 2005; Yuan et al., 2011), (2) a parent‐specific methylation pattern in gametes of an oviparous fish (suggesting that the foundations of genomic imprinting also exist in teleost fish; Xie et al., 2009), and (3) the evolution in fish of the manose‐6‐phosphate receptor into an insulin‐like growth factor 2 receptor (*igf2r*) with a role on *igf2* degradation (Nolan et al., 2006) that has a similar structure and affinity for IGF2 to that of the mammalian gene (Méndez et al., 2001), suggests that the possibility of genetic imprinting in this group of viviparous vertebrates should be investigated.

Paternal manipulation in developing offspring may be countered by maternal adaptations to mitigate its effects. If this antagonistic coevolution is not completely matched in isolated populations, asymmetric embryonic growth of the type that we detected in the interpopulation crosses may occur (although we did not find a substantial decrease in embryo size in M‐Z broods, which would provide evidence of co‐evolved female resistance to any manipulation by the males; see Moore & Haig, 1991).

A parent‐of‐origin *igf2* methylation pattern in *G. multiradiatus* may be the consequence of several processes, including epigenetic regulation as that seen in mammals (Lawton et al., 2008; Murrell, Heeson, & Reik, 2004). Our data indicate that *G. multiradiatus* females from different populations produce offspring of different size, but do not modify the number of offspring per brood, when mated with allopatric males. This could happen if maternal factors were differentially at play and is also consistent with male manipulation of female reproductive allotment; and experimental manipulation is required to tease these possibilities apart.

The sexually antagonistic IGF system is only known to occur in mammals, but its constitutive elements are found in fish, raising the possibility that it evolved independently in mammals and teleosts, or that it was present in the ancestors of the two lineages diverged. Previous efforts to demonstrate imprinting of *igf2* in placental Poeciliid species have been unsuccessful (Lawton et al., 2005); yet, we found evidence that suggest parent‐of‐origin gene expression in the Goodeidae (which are also cyprinodontids). Some attributes that may favor the evolution of a genetic antagonistic coevolution mediated by IGF2 in the Goodeidae include enforceable female mate choice. This may be linked to the fact that goodeid embryos’ dry weight can increase up to 38,700% (*Zoogoneticus quitzeoensis;* Wourms et al., 1988; Hollenberg & Wourms, 1995), whereas placental poeciliid embryos achieve at most 11,700% (*P. retropinna*; Wourms 1981). Such greater mass increase takes place during a gestation period that lasts about 8 weeks; twice as much that of poeciliids. We think that the massive reproductive allocation of goodeid females, together with the existence of a trophotaenial placenta (a fetal structure involved in the capture and transport of nutrients from the ovarian lumen/walls to the embryonic gut; Lombardi & Wourms, 1985a,b), provides both the opportunity and the physiological conditions in which *igf2* can influence maternal investment.

The breeding system of the Amarillo (*Girardinichthys multiradiatus*) fits the conditions stipulated by Wilkins and Haig (2003) as potential promoters of genomic imprinting: (1) Broods can be sired by more than one male (Macías‐Garcia & Saborío, 2004), (2) females bear the bulk of the reproductive costs (e.g., Lombardi & Wourms, 1985a,b), and (3) their allocation of resources can be influenced by genes that are expressed in the embryos (e.g., *igf2*; see O'Neill et al., 2007); therefore, further research on the possibility of genomic imprinting of *igf2* of this fish is needed.

## CONFLICT OF INTERESTS

All authors declare that we do not have any competing financial interests.

## AUTHOR CONTRIBUTIONS

YSL carried out most of the experiments and analyses and wrote the first draft of the manuscript in conjunction with CMG. J‐PV‐C provided logistical, technical, and financial support. MGR provided logistical and technical support. CMG provided logistical support and wrote the first draft of the manuscript in conjunction with YSL. All authors contributed to the design of the experiments, the interpretation of the results, and the edition of the manuscript.
