# Supplementary material for: Asymmetric paternal effect on offspring size linked to parent‐of‐origin expression of an insulin‐like growth factor
Source: Ecol Evol. 2017 May 15;7(12):4465–74. doi: 10.1002/ece3.3025 (PMC5478053; doi:10.1002/ece3.3025)
Supplement: Supplementary file 1 [file ECE3-7-4465-s001.docx]

**Appendix**

**Figure S1. Scheme of *igf2***

**Figure S2. Female reproductive allocation and brood size**

**Table S1. Complementary statistical data on reproductive allocation**

**Table S2. List of primers**

**Table S3. Complementary statistical data on offspring standard length**

**Table S4. Complementary statistical data on offspring width**

**Table S5. List of the families that were genotyped for the parent of origin *igf2* expression experiment**

**Methods**

1. **Parent of origin *igf2* expression**
2. **DNA extraction protocol.**

**Use of near-terminus embryos**

***Igf2* sequencing.**

***Igf2* allelic expression**

**Figure S1.** Representation of *igf2* of *G. multiradiatus* and the primers that were used for sequencing*.* Blue boxes represent the exons and continuous lines the introns. Green arrowheads show the binding site of the primers used for the amplification of the ≈ 5-kb fragment. Purple arrowheads show the binding site of the internal primers used for sequencing *igf2.*

**
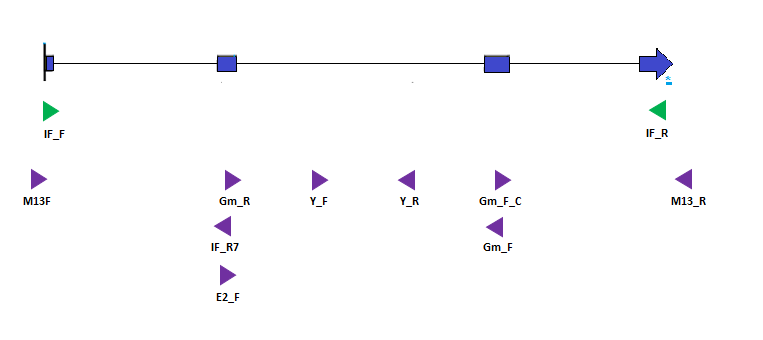
**

**Figure S2.** a) Reproductive allocation by female *G. multiradiatus* from San Matías (M) and Zempoala (Z) was the same when mated to Zempoala, but not to San Matias males (the effect was not significant after Bonferroni; see below). Zempoala females produced larger broods irrespective of male origin (b). Graphs show means adjusted for female size (SL).


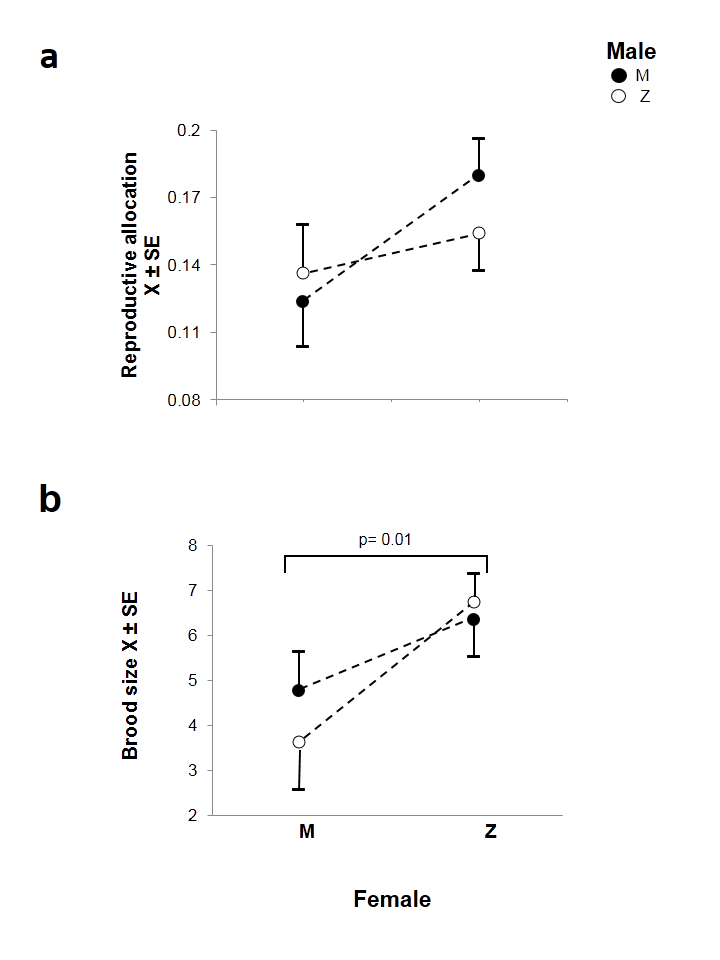


|  | Mean | |  | DF | |  | | | P | | |
| --- | --- | --- | --- | --- | --- | --- | --- | --- | --- | --- | --- |
| Comparison | difference | F | | numerator | denominator | |  | raw | | | Bonferroni |
| **Mother** |  | 3.192 | | 1 | 52 |  | | | 0.080 |  | |
| ♀ M - Z | -0.0369 | 3.192 | | 1 | 52 |  | | | 0.080 | 0.080 [1] | |
|  |  |  | |  |  |  | | |  |  | |
| **Father** |  | 0.121 | | 1 | 52 |  | | | 0.729 |  | |
| ♂ M - Z | 0.0063 | 0.121 | | 1 | 52 |  | | | 0.729 | 0.729 [1] | |
|  |  |  | |  |  |  | | |  |  | |
| **Rearing environment** |  | 3.370 | | 1 | 52 |  | | | 0.072 |  | |
| 1-2 pairs – communal | 0.0358 | 3.370 | | 1 | 52 |  | | | 0.072 | 0.072 [1] | |
|  |  |  | |  |  |  | | |  |  | |
| **Mother x Father** |  | 1.122 | | 1 | 52 |  | | | 0.294 |  | |
| ♀ = M, ♂: M - Z | -0.0129 | 0.207 | | 1 | 52 |  | | | 0.651 | 1.000 [2] | |
| ♀ = Z, ♂: M - Z | 0.0255 | 1.265 | | 1 | 52 |  | | | 0.266 | 0.532 [2] | |
|  |  |  | |  |  |  | | |  |  | |
| ♂ = M, ♀: M - Z | -0.0561 | 4.581 | | 1 | 52 |  | | | **0.037** | 0.074 [2] | |
| ♂ = Z, ♀: M - Z | -0.0177 | 0.383 | | 1 | 52 |  | | | 0.539 | 1.000 [2] | |
|  |  |  | |  |  |  | | |  |  | |
| Covariate: female SL=31.2753 | |  | |  |  |  | | |  |  | |

Table S1.Results from the mixed GLM on data presented in Figure S2 (above).

**Table S2**. Sequence of the primers used to amplify different fragments of *igf2.*

| Primer | Sequence |
| --- | --- |
| ***igf2* amplification** |  |
| IF_F | 5’ ATGGAGACCCAGCAAAGATCCGGAC 3’, |
| IF_R | 5’ AATTGTCTGTGGTGTGCAACACGGC3’ |
| **Gene sequencing** |  |
| IF_R7 | 5’ CAGACAAACTGCAGCGCATC 3’ |
| GM_R | 5’ GAAATGGCCTCGGCAGAGACGTGGT 3’ |
| GM_F | 5’ TTGCTCCAGCAGGTTGAGGTCACAG 3’ |
| GM_F_C | 5’ CCGTGGGATTGTAGAAGAGTG 3’ |
| Y_F | 5’ ATGAGCTGGGAGGAACGAAA 3’ |
| Y_R | 5’ AGG GAG AGG GAG GAC TGA TT 3’ |
| E2_F | 5’ TGCTTTAATTTGTGTGTTTCCCC 3’ |
| **Genotyping** |  |
| I1_F2_P | 5’ GAGTTACCAGGTCAGTGCGT 3’ |
| I2_R2_P | 5’ AGAGGAAAGGGGAGCGAAAA 3’ |
| **RTPCR** |  |
| Gm_A_F | 5’ GGAGACCCAGCAAAGATCCG 3’ |
| IF_R7  **Bisulfite sequencing**  Forward 1  Forward 2  Reverse 1  Reverse 2 | 5’ GATGCGCTGCAGTTTGTCTG 3’  5'GAGTTACCAGGTCAGTGCGT3'  5’GAGTTATTAGGTTAGTGTGT3’  5'AGAGGAAAGGGGAGCGAAAA3’  5’AGAGGAAAGGGGAGTGAAAA3’ |

**Table S3.** Output from the Generalized Mixed Model (GML) used to evaluate differences in offspring standard length (SL) between crosses. Mother = population of origin of the mother, Father = population of origin of the father, SL = Standard Length, M = San Matías, Z = Zempoala. Output generated by NCSS 2007 v.7.1.21.

**Term-by-Term Hypothesis Test Results**

**Model Num. Denom.**

**Term *F-*Value *df* *df* *p -* Level**

Female SL 23.3453 1 61.4 0.000009

Mother 0.0767 1 64.5 0.782744

Father 1.0561 1 64.9 0.307921

Mother*Father 7.4564 1 64.4 0.008146

Rearing environment 0.6033 1 65.0 0.440152

**Standard**

**Error**

**Name Mean of Mean**

**Intercept**

**Mother*Father**

M, M 11.0218 0.2595

M, Z 11.4388 0.2948

Z, M 11.6107 0.2368

Z, Z 10.7004 0.2037

**Individual Comparison Hypothesis Test Results**

**Covariates: Female SL=32.7863**

**Comparison**

**Comparison/ Mean Num. Denom. Raw Bonferroni**

**Covariate(s) Difference *F-*Value *df* *df* *p* - Level *p* - Level**

Mother 0.0767 1 64.5 0.782744

Mother: M - Z 0.0747 0.0767 1 64.5 0.782744 0.782744 [1]

Father 1.0561 1 64.9 0.307921

Father: M - Z 0.2466 1.0561 1 64.9 0.307921 0.307921 [1]

Mother*Father 7.4564 1 64.4 0.008146

Mother = M, Father: M - Z -0.4170 1.1665 1 65.6 0.284069 0.568139 [2]

Mother = Z, Father: M - Z 0.9103 9.8317 1 63.0 0.002606 0.005211 [2]

Father = M, Mother: M - Z -0.5889 2.7558 1 66.4 0.101616 0.203232 [2]

Father = Z, Mother: M - Z 0.7384 3.9518 1 62.8 0.051188 0.102376 [2]

Rearing environment 0.6033 1 65.0 0.440152

1-2 pairs - communal -0.1914 0.6033 1 65.0 0.440152 0.440152 [1]

These *F-*Values test Type-III (adjusted last) hypotheses.

**Report Definitions**

Comparison/Covariate(s): Illustrates the comparison being made.

Comparison Mean Difference: The difference in means for each comparison.

*F-*Value: The test statistic value corresponding to the L matrix used for testing the model term.

Num *DF*: The numerator degrees of freedom for the *F-*test.

Denom *DF*: The denominator degrees of freedom for the *F-*test.

Raw Prob Level: Gives the strength of evidence for a single comparison, unadjusted for multiple testing.

Bonferroni Prob Level: Gives the p-value adjusted to multiple tests. The number in brackets (e.g. [4]) denotes the number of tests for which the raw prob level was adjusted.

**Table S3.** Output from the Generalized Mixed Model (GML) used to evaluate differences in offspring width between crosses. Mother = population of origin of the mother, Father = population of origin of the father, SL = Standard Length, M = San Matías, Z = Zempoala. Data obtained from the output generated by NCSS 2007 v.7.1.21.

**Term-by-Term Hypothesis Test Results**

**Model Num. Denom.**

**Term *F-*Value *df* *df* *p -* Level**

Female SL 15.0314 1 62.3 0.000257

Mother 0.9351 1 65.2 0.337123

Father 1.1833 1 65.5 0.280661

Mother*Father 4.5083 1 65.2 0.037533

Rearing environment 0.5192 1 65.3 0.473762

**Standard**

**Error**

**Name Mean of Mean**

**Intercept**

**Mother*Father**

M, M 2.5326 0.0895

M, Z 2.6209 0.1020

Z, M 2.6210 0.0824

Z, Z 2.6210 0.0824

**Individual Comparison Hypothesis Test Results**

**Covariates: Female SL=32.7863**

**Comparison**

**Comparison/ Mean Num. Denom. Raw Bonferroni**

**Covariate(s) Difference *F-*Value *df* *df* *p* - Level *p* - Level**

Mother 0.9351 1 65.2 0.337123

Mother: M - Z 0.0904 0.9351 1 65.2 0.337123 0.337123 [1]

Father 1.1833 1 65.5 0.280661

Father: M - Z 0.0904 1.1833 1 65.5 0.280661 0.280661 [1]

Mother*Father 4.5083 1 65.2 0.037533

Mother = M, Father: M - Z -0.0884 0.4377 1 66.4 0.510520 1.000000 [2]

Mother = Z, Father: M - Z 0.2692 7.1379 1 63.5 0.009577 0.019154 [2]

Father = M, Mother: M - Z -0.0884 0.5192 1 66.8 0.473716 0.947433 [2]

Father = Z, Mother: M - Z 0.2692 4.3613 1 63.8 0.040761 0.081522 [2]

Rearing envinronment 0.5192 1 65.3 0.473762

1-2 pairs – communal -0.0615 0.5192 1 65.3 0.473762 0.473762 [1]

These *F-*Values test Type-III (adjusted last) hypotheses.

**Report Definitions**

Comparison/Covariate(s): Illustrates the comparison being made.

Comparison Mean Difference: The difference in means for each comparison.

*F-*Value: The test statistic value corresponding to the L matrix used for testing the model term.

Num *DF*: The numerator degrees of freedom for the *F-*test.

Denom *DF*: The denominator degrees of freedom for the *F-*test.

Raw Prob Level: Gives the strength of evidence for a single comparison, unadjusted for multiple testing.

Bonferroni Prob Level: Gives the p-value adjusted to multiple tests. The number in brackets (e.g. [4]) denotes the number of tests for which the raw prob level was adjusted.

**Table S4.** Analysis of the sequence of exon 2 of *igf2* of 22 families of G. multiradiatus. Z = Zempoala, H = Huapango T = Tonatiahua. T and Z are mountain lakes separated by less than 0.5 Km. Crosses that could have been used for the parent of origin methylation pattern are marked with an *.

| **Family** | **Female ID** | **Male ID** | **Number of female clones sequenced** | | | **Number of male clones sequenced** | | | **Brood size** |
| --- | --- | --- | --- | --- | --- | --- | --- | --- | --- |
|  |  |  | **T** | **C** | **Total** | **T** | **C** | **Total** |  |
| 1 | 1Z | 1Z | 9 | 0 | 9 | 9 | 0 | 9 | NR |
| 2* | 2T | 1Z | 7 | 2 | 9 | 9 | 0 | 9 | 7 |
| 3 | 3Z | 3Z | 7 | 0 | 7 | 11 | 0 | 11 | 5 |
| 4 * | 4Z | 4Z | 4 | 1 | 5 | 10 | 0 | 10 | 6 |
| 5 | 7H | 4Z | 9 | 0 | 9 | 10 | 0 | 10 | 3 |
| 6 | 8Z | 6Z | 7 | 0 | 7 | 4 | 0 | 4 | 2 |
| 7 | 9H | 7Z | 8 | 0 | 8 | 8 | 0 | 8 | 5 |
| 8 | 10Z | 8Z | 10 | 0 | 10 | 8 | 0 | 8 | 9 |
| 9 | 14Z | 8Z | 9 | 0 | 9 | 8 | 0 | 8 | 10 |
| 10 | 12Z | 10Z | 4 | 0 | 4 | 4 | 6 | 10 | 3 |
| 11 | 23Z | 10Z | 5 | 5 | 10 | 4 | 6 | 10 | 11 |
| 12 | 13Z | 11Z | 10 | 0 | 10 | 9 | 1 | 10 | 9 |
| 13 | 21H | 11Z | 8 | 2 | 10 | 9 | 1 | 10 | 10 |
| 14 | 22Z | 11Z | 6 | 4 | 10 | 9 | 1 | 10 | 8 |
| 15 | 15Z | 13Z | 10 | 0 | 10 | 10 | 0 | 10 | 7 |
| 16 | 16Z | 14Z | 7 | 3 | 10 | 1 | 0 | 1 | 14 |
| 17* | 17Z | 15Z | 6 | 4 | 10 | 7 | 0 | 7 | 3 |
| 18 | 18Z | 16Z | 10 | 0 | 10 | 5 | 0 | 5 | 7 |
| 19 | 19H | 16Z | 8 | 0 | 8 | 5 | 0 | 5 | 7 |
| 20 | 20H | 17Z | 5 | 5 | 10 | 4 | 7 | 11 | 11 |
| 21 | 24Z | 18Z | 12 | 0 | 12 | 7 | 3 | 11 | 9 |
| 22 | 25Z | 19Z | 9 | 1 | 10 | 9 | 1 | 10 | 13 |

NR. Not recorded

Table S5. Genotyping adults and offspring for the informative SNP. Thirty six individuals were genotyped. Only five fish from Huapango and one fish from Tonatiahua were used for the crosses, the other 30 individuals being from Zempoala. We did not find any homozygous CC individuals. We genotyped 6 offspring of one C/T x C/C cross (family 4) and used two heterozygous offspring P4-1 and P4-5 for the parent of origin *igf2* expression assay.

Numbers of adults that were genotyped

| **T/T** ♀ | | |  | **T/T** ♂ | | |  | **T/C** ♀ | | |  | **T/C** ♂ | | |  | **C/C** ♀ | | |  | **C/C** ♂ | | |
| --- | --- | --- | --- | --- | --- | --- | --- | --- | --- | --- | --- | --- | --- | --- | --- | --- | --- | --- | --- | --- | --- | --- |
| 13 | | |  | 9 | | |  | 9 | | |  | 5 | | |  | 0 | | |  | 0 | | |
| Z  10 | T  0 | H  3 |  | Z  9 | T  0 | H  0 |  | Z  6 | T  1 | H  2 |  | Z  5 | T  0 | H  0 |  | Z  0 | T  0 | H  0 |  | Z  0 | T  0 | H  0 |

Parent of origin *igf2* expression of a C/T x C/C cross

|  | **Genotyping** | | |  | **RT-PCR** | | |
| --- | --- | --- | --- | --- | --- | --- | --- |
| Offspring ID | Number of clones | | |  | Number of clones | | |
|  | Paternal T allele | Maternal C allele | Total |  | Paternal T allele | Maternal C allele | Total |
| P4-1 | 64T | 4C | 68 |  | 78T | 0C | 78 |
| P4-2 | 17T | 0C | 17 |  |  |  |  |
| P4-3 | 7T | 0C | 7 |  |  |  |  |
| P4-4 | 20T | 0C | 20 |  |  |  |  |
| P4-5 | 18T | 1C | 19 |  | 54T | 0C | 54 |
| P4-6 | 20 T | 0C | 20 |  |  |  |  |

Z= Zempoala, T=Tonatiahua, H= Huapango.

**Methods**

1. **Parent of origin *igf2* expression and bisulfite sequencing**

Fish husbandry

Individuals were either offspring of adult fish collected from the field, or adults from the field collected as above (SAGARPA permit SGPA/DGVS/01290/13). Broods were kept separately in 20L aquaria, and as soon as the sex of the juveniles could be identified we formed couples or trios using fish from different broods, and kept at of 12-hour-day-night cycle, 21ºC and fed SeraVipan commercial fish flakes twice a day

1. **DNA extraction protocol**

# **Cell Lysis solution** **Others:**

0.1M EDTA 20mg/ml Proteinase K

0.2M Tris pH 8.5 5 M Potassium Acetate

1% SDS 10mg/ml Rnase A

Isopropanol

70% Ethanol

Distilled H_2_0 or TE

# **Protocol**

1. Chill a 1.5ml tube containing 600ul Cell Lysis solution on ice (may turn cloudy).
2. Add 10-20 mg of fresh or frozen tissue to the solution. Remove from ice and quickly homogenise with a microfuge tube pestle. Keep on ice.
3. Incubate at 55^0^C for 15-60 minutes, for maximum yield add 3ul of Proteinase K and incubate at 55^0^C overnight.
4. Add 3ul of RNase A
5. Mix by inverting tube many times at 37^0^C for 15-60 mins and put them on ice.
6. Add 200ul 5M KAc.
7. Put the tubes on ice for five minutes and vortex vigorously at high speed for 20 seconds
8. Centrifuge top speed in microfuge for 5 mins at 4ºC to precipitate proteins. The precipitated proteins should form a tight pellet, if not then re-vortex for 20 seconds to mix sample and incubate on ice for 5 mins. Re- centrifuge top speed for 3 mins.
9. Decant supernatant containing DNA into a clean 1.5ml eppendorf tube containing 600ul of cold (from freezer) 100% isopropanol. Mix the sample until DNA clumps form. 1/10^th^ volume of 3M NaAc, pH5.2 can be added to help precipitation of DNA. DNA can be left at -80 for 1hr or overnight.
10. Centrifuge at top speed for 2 min at 4ºC.
11. Pour off supernatant and drain tube on clean absorbent paper. Add 600ul of 70% ethanol to wash pellet. Centrifuge for 1 min at 4ºC, pour off alcohol, drain then REPEAT AGAIN.
12. Centrifuge top speed in microfuge for 1 min.
13. Pipette off any residual ethanol and air dry for approx. 20 mins – don’t leave drying too long or DNA becomes difficult to re suspend. (all ethanol should be gone before re suspending)
14. Re suspend in TE or H_2_0 (PREFFERED FOR PCR). The DNA may require heating to re suspend, heat at 65 degrees for 1 hour, tap tube periodically to aid in dispersing the DNA.
15. Store DNA samples in freezer -20 degrees long term.

**Use of near-terminus embryos.**

We used near-terminus embryos for the assessment of allelic expression. It has been shown in another matrotrophic fish (*Heterandria formosa*) that *IGF2* synthesis is higher in the early stages of embryonic development (Schrader & Travis 2012), yet we were constrained to do this because pregnancy is not guaranteed in small social groups in laboratory (56 % for intra- and 24 % for inter-population crosses when females are kept with a single male, González Zuarth & Macías Garcia 2006), and early pregnancy is undetectable, thus we had to wait until it was evident rather than unnecessarily sacrificing many females.

***igf2* sequencing**

Genomic DNA was isolated from two male-female pairs, one from Zempoala and one from San Matías, using Qiagen DNeasy Blood & Tissue Kit.

Using the sequence of *igf2* of *Ilyodon ameca* (GenBank Accession number DQ337453.1) as a reference, we designed primers (Table S1) that flank virtually all the ≈5 kb Open Reading Frame, except for the last 17 nucleotides, of *G. multiradiatus igf2*. (Figure S1). The amplification PCR reaction system contained 16 µl of PCR SuperMix High Fidelity (Invitrogen), 0.5 µl IF_F (50 ng/µl), 0.5 µl IF_R (50 ng/µl), 1 µl DNA, and was put through a cycling profile of 95 ºC for 5 min, 94 ºC for 30 s, 62 ºC for 30 s, 68 ºC for 5:30 min, 30 cycles, and then 68 ºC for 10 min.

To clone the ORF fragment we followed the protocol of TOPO TA Cloning (Invitrogen) for electrocompetent cells (TOP10 Electrocomp). Plasmid DNA was extracted using the alkaline lysis protocol by Sambrook et al. (1989). The 5 kb fragment was sequenced using several overlapping internal primers (Table S1, Figure S1). Five sequences of each individual were assembled and screened for SNPs.

The *igf2* gene of *G. multiradiatus* is composed of four exons and three introns, which together have an ORF ≈ 5 kb. We sequenced 4814 nucleotides, which included exons 1, 2 and 3, introns 1, 2 and 3, and nearly all the sequence of exon 4, barring the last 17 nucleotides. The coding region of *igf2* was virtually identical in the fish from both populations, but we identified five potential SNPs in exon 2 and two in exon 4. Exon 2 had more alleles than exon 4, so we used the former for the allelic expression experiments. None of the putative SNPs were exclusive to one population; however, fish from Zempoala were more polymorphic than those from San Matias, and one SNP was sufficiently frequent among the Z-fish to be used for screening of allelic expression. Thus, we selected the Zempoala population to determine whether *igf2* is expressed monoallelically or biallelically in *G. multiradiatus.*

***Igf2* allelic expression**

To determine the genotypes of *igf2* transcripts present in developing embryos, we conducted RT-PCR of the *igf2* gene by amplifying a 201 bp cDNA fragment spanning the SNP. The cross analysed was between a homozygous T/T male and a heterozygous C/T female, so half the offspring should have been heterozygous. The family included two heterozygous offspring out of six. *igf2* RT-PCR amplification yielded 78 and 54 independent cDNA fragments from these two individuals that were sequenced after cloning. The sequence of all recovered cDNAs corresponded to only the paternally inherited allele, suggesting that the maternal allele is not expressed in the developing embryos (Table S5); nonetheless, this assessment of parent of origin expression is rather provisional given the scarcity of heterozygous fish.
